# Analysing the performance of low-cost air quality sensors, their drivers, relative benefits and calibration in cities—a case study in Sheffield

**DOI:** 10.1007/s10661-019-7231-8

**Published:** 2019-01-22

**Authors:** Said Munir, Martin Mayfield, Daniel Coca, Stephen A. Jubb, Ogo Osammor

**Affiliations:** 1 Department of Civil and Structural Engineering, The University of Sheffield, Sheffield, S1 3JD UK; 2 Department of Automatic Control and Systems Engineering, University of Sheffield, Sheffield, S1 3JD UK; 3 Air Quality Monitoring & Modelling, Sheffield City Council, Howden House, 1 Union Street,, Sheffield, S1 2SH UK

**Keywords:** Sensor cost, Sensor networks, Envirowatch E-MOTEs, Air pollution monitoring, Generalised additive model

## Abstract

Traditional real-time air quality monitoring instruments are expensive to install and maintain; therefore, such existing air quality monitoring networks are sparsely deployed and lack the measurement density to develop high-resolution spatiotemporal air pollutant maps. More recently, low-cost sensors have been used to collect high-resolution spatial and temporal air pollution data in real-time. In this paper, for the first time, Envirowatch E-MOTEs are employed for air quality monitoring as a case study in Sheffield. Ten E-MOTEs were deployed for a year (October 2016 to September 2017) monitoring several air pollutants (NO, NO_2_, CO) and meteorological parameters. Their performance was compared to each other and to a reference instrument installed nearby. E-MOTEs were able to successfully capture the temporal variability such as diurnal, weekly and annual cycles in air pollutant concentrations and demonstrated significant similarity with reference instruments. NO_2_ concentrations showed very strong positive correlation between various sensors. Mostly, correlation coefficients (*r* values) were greater than 0.92. CO from different sensors also had *r* values mostly greater than 0.92; however, NO showed *r* value less than 0.5. Furthermore, several multiple linear regression models (MLRM) and generalised additive models (GAM) were developed to calibrate the E-MOTE data and reproduce NO and NO_2_ concentrations measured by the reference instruments. GAMs demonstrated significantly better performance than linear models by capturing the non-linear association between the response and explanatory variables. The best GAM developed for reproducing NO_2_ concentrations returned values of 0.95, 3.91, 0.81, 0.005 and 0.61 for factor of two (FAC2), root mean square error (RMSE), coefficient of determination (*R*^2^), normalised mean biased (NMB) and coefficient of efficiency (COE), respectively. The low-cost sensors offer a more affordable alternative for providing real-time high-resolution spatiotemporal air quality and meteorological parameter data with acceptable performance.

## Introduction

With an increasing trend towards urbanisation due to better job opportunities and greater access to amenities and facilities in cities, urban areas are expanding rapidly globally. Given this trend, air pollutant levels are increasing, especially in large urban agglomerations and at roadside locations, which adversely impact human health in a variety of ways. Air pollutants, especially high levels of nitrogen dioxide (NO_2_) and particulate matter (PM_10_ and PM_2.5_) are considered the most significant environmental risks to public health in urban areas in the UK (Department for Environment, Food and Rural Affairs (DEFRA) 2015; World Health Organisation (WHO) 2013). Atmospheric air pollutants were estimated to cause seven million premature deaths in 2012, worldwide (WHO 2014). Air pollutants (e.g. NO_2_ and PM_10_) emitted by various emission sources are risk factors and are reported to increase the risk of incidence of various diseases including heart disease, lung cancer and both chronic and acute respiratory diseases, including asthma (WHO 2014).

Air quality monitoring is important to promote air quality awareness and to support abatement strategies (Borrego et al. 2016). Several techniques are used to monitor air quality (Penza et al. 2014), which include (a) Reference or conventional real-time air quality monitoring, (b) portable air quality monitors, (c) passive diffusion tubes and (d) digital sensors. Reference air quality monitoring instruments are the most accurate and are used for air quality compliance purposes, studying exposure, supporting air quality management and developing policies for reducing and controlling emissions. Reference instruments are expensive to purchase and maintain, and therefore, the spatial resolution of air quality measurement is low and insufficient for detailed spatiotemporal mapping. Portable or mobile monitors are either carried by individuals or installed in vehicles that can be stationed where fixed continuous monitors cannot be installed. Portable instruments can be useful for monitoring air quality in certain cases and can provide high-resolution temporal data for a short period of time, but have limited application for spatial mapping and long-term monitoring. Passive tubes are small collection devices used for monitoring gaseous air pollutants such as NO_2_ and typically provide monthly average concentrations, which can be converted to annual averages. These diffusion tubes are the cheapest technique and provide better spatial coverage. However, these can be used only for gaseous air pollutants and for long-term monitoring (mainly monthly average). Low-cost sensors (LCS) are used to collect real-time air quality data providing high-resolution spatial and temporal air quality data. These type of sensors are the new trend in air quality monitoring and can support the conventional air quality monitoring stations to increase the density of the sensing network (Heimann et al. 2015; Van den Bossche et al. 2015; Viana et al. 2015). The low-cost sensors use the latest microsensing technology and are considered the innovative tools for air quality monitoring in the future (Castell et al. 2015; Snyder et al. 2013; Kumar et al. 2015; Stojanovic et al. 2015). Data collected by these sensors can be used for detailed spatial and temporal mapping of air pollution, especially over distinct areas such as city or an urban district, for atmospheric model validation and assessing population exposure; however, the data need to be handle with caution and several corrections need to be applied first.

Several authors have analysed the performance of the LCS, comparing their performance with reference instruments and with each other. Borrego et al. (2016) performed such an assessment (sensors compared to reference instruments) in Aveiro, Portugal, from 13 to 27 October 2014. The LCS and reference instruments were colocated and monitored the levels of gaseous pollutants (e.g. CO, NOx, O_3_, SO_2_), particulate matter (PM_10_, PM_2.5_) and meteorological parameters (e.g. temperature, wind speed and direction, relative humidity, solar radiation and precipitation). The resultant measurements were mutually compared and different sensors showed significantly different performance in terms of the statistical metrics used for evaluating the sensors’ performance. The range of *R*^2^ (coefficient of determination) values for different air pollutants was O_3_ (0.12–0.77), CO (0.53–0.87), NO_2_ (0.02–0.89), PM (0.07–0.36) and SO_2_ (0.09–0.20), where a lower *R*^2^ value shows poor measurement performance of the sensors. Borrego et al. (2016) concluded that LCS had great potential for air quality monitoring, if properly supported by post-processing and data modelling tools.

Different sensor systems use different principles to measure the concentrations of atmospheric pollutants (Borrego et al. 2016). These include optical particle counters (OPC), metal oxide semiconductor sensors (MOS), electrochemical sensors (EC), non-dispersive infrared sensors (NDIR) and photo-ionisation detection sensors (PID). Aleixandre and Gerboles (2012) reported that these air quality sensors work through either measuring the electrochemical interaction between the sensing materials and the atmospheric chemicals or through absorption of visible light. The principle of light scattering or absorption is used for measuring the levels of PM. Individual sensors are usually integrated into a platform of sensors known as a sensor node. Each sensor node contains a sensor board, the sensors and a control board which integrates all the elements of the hardware such as GPS, data storage, communication ports and signal conditioning. Examples of networks based on these types of sensors are (a) Cambridge University Sensor Network for Air Quality (SNAQ) (Mead et al. 2013; Popoola et al. 2013; Borrego et al. 2016), (b) AUTh-ISAG AQ Microsensors (Borrego et al. 2016), (c) Energy Centre of Netherlands (ECN Airbox) (Borrego et al. 2016; Hamm et al. 2016), (d) NanoEnvi platform (Borrego et al. 2016), (e) AQMesh sensors (Borrego et al. 2016; Carruthers et al. 2016), (f) ENEA Air-Sensor (Suriano et al. 2015), (g) EveryAware Sensor Box (Borrego et al. 2016) and Envirowatch E-MOTE sensors (Reis et al. 2013). These sensors are briefly described below.

- Cambridge university SNAQ are microsensors for measuring the concentrations of multispecies including gases air pollutants, particulate matter and meteorological parameters. These are low-cost sensors and can be powered by battery or mains. Mead et al. (2013) employed these microsensors for monitoring air quality in Cambridge. Static sensors were deployed to street furniture, whereas mobile sensors were carried by pedestrians and cyclists. Mead et al. (2013) reported widely varying concentrations of air pollutants in the urban environment, which could not be characterised by sparse static conventional air quality network. Furthermore, Popoola et al. (2013) deployed these sensors in Heathrow Airport in London for air quality monitoring. They reported considerable spatial and temporal variations in air pollutant concentrations across the air quality network. According to their findings, high air pollutant levels were linked with stable weather conditions.
- AUTh-ISAG AQ Microsensors use the principle of Waspmote wireless network, developed by Libelium, which is an international IT and engineering company. These sensors aim to reduce power consumption, reduce thermal noise, provide easy inspection and require low maintenance. Data are normally collected using an SD card and can be run using both battery and main power supply. These sensors were used by Borrego et al. (2016) in their study and their performance was compared to several other microsensors and reference instruments. These sensors can measure the concentrations of several air pollutants and meteorological parameters.
- ECN Airbox were developed by the Energy Research Centre of the Netherlands (ECN). Airbox sensors monitor particulate matter (e.g. ultrafine particles (UFP), PM_1_, PM_2.5_ and PM_10_), gaseous (e.g. NO_2_ and O_3_) and meteorological parameters (e.g. temperature and relative humidity). Airbox sensors have been used for air quality monitoring in the Netherlands in the city of Eindhoven in 35 locations since 2013. These sensors are powered by battery and mains. Hamm et al. (2016) have provided a detailed review of these sensors, which could be read for further details.
- NanoEnvi sensors were manufactured by Envira. These analysers use several sensors with different technology. The sensors’ work is based on the changes in electrical properties that happen in the surface of the sensors when pollutants are present. The air pollutants which can be measured by NanoEnvi are gaseous pollutants (e.g. SO_2_, NO, NO_2_, CO, CO_2_, O_3_, H_2_S and VOCs), particulates (PM_10_ and PM_2.5_) and meteorological parameters (e.g. wind characteristics, temperature, relative humidity).
- AQMesh sensors are manufactured by Environmental Instruments Ltd., UK. These are low-cost microscale sensors for effective environmental monitoring, which are developed for harsh outfield environmental conditions and are capable of working to high standards. AQMesh microsensors measure the concentrations of NO, NO_2_, O_3_, SO_2_ and CO using the latest generation of electrochemical sensors. Particulate matter is measured using a light scattering optical particle counter. Using solid state sensors, they can also measure the levels of temperature, RH and atmospheric pressure. Carruthers et al. (2016) compared the performance of AQMesh in Cambridge with reference instruments where AQMesh showed considerably higher concentrations of NO_2_, NO and PM_10_; however, overall, they performed well and showed great potential for contributing to the air quality monitoring, especially improving the spatial coverage in the UK.
- ENEA Air-Sensor are manufactured by ENEA (Energia Nucleare ed Energie Alternative), which is an Italian agency for new technology, energy and environment. These sensors measure the levels of several air pollutants, such as CO, NO_2_, O_3_, SO_2_, H_2_S and PM_10_, and meteorological parameters such as relative humidity and temperature. These sensors can be operated via battery or mains. Suriano et al. (2015) evaluated the performance of these air sensors during a campaign of several months in Italian national projects for sustainable innovation in the smart cities. These sensors were used both as stationary and mobile air quality monitoring systems, and initial results indicated that these sensors potentially could improve air quality monitoring program.
- EveryAware sensors are manufactured by Vito (a leading independent research and technology organisation based in Belgium and works in the areas of cleantech and sustainable development) under the European Seven Framework Program (EU-FP7). The EveryAware sensors are used for air quality monitoring in Belgium, Italy and the UK. EveryAware is a low-cost, portable air quality monitor used for measuring personal exposure to traffic pollution. This device contains six low-cost gas sensors that react in the presence of traffic pollutants (e.g. CO, NOx). Borrego et al. (2016) used EveryAware sensors in Aveiro, Portugal, to compare their performance with other microsensor and reference instruments.

Dongol (2015) has listed several sensor platforms which include DunavNet Platform, UrVamm, GeoTech and ATEKNEA. In addition to these sensors, there are several other types of sensors available for air quality monitoring and the listing is growing with time. Sensors of this type are cheaper, compact, user-friendly and provide high-resolution spatiotemporal air pollutant concentrations. They have the potential to enhance the existing air quality network run at local levels by local authorities and nationally by DEFRA. In addition, these sensors can be installed independently by various research and governmental organisations to monitor public exposure to various air pollutants within a specific area. Despite all these positive points, the quality of air pollution data collected by these sensors is unproven and cannot be used for regulatory and compliance purposes; however, the data can be used for highlighting air pollution hotpots, for public awareness and for complementing traditional air quality monitoring programmes. There is a need for further investigation to quantify uncertainties in the datasets these types of sensors produce. These uncertainties are related to exposure to harsh environmental conditions, especially extreme temperature and relative humidity and the associated time interval (i.e. the length of time the instruments are operated in such a harsh environment). Furthermore, uncertainties are also affected by the measuring principles of the sensors and the quality of the materials used by the manufacturers. Therefore, inter-comparison of LCS made by different manufacturers and with reference instruments is required. Further work is also required to improve the performance of these sensors by (a) improving their technology further to make it more robust, (b) frequent calibration both in laboratory and outdoor and (c) improving the experimental designs.

In this project, the aim is to install LCS in the city of Sheffield to provide high-resolution spatiotemporal maps of various air pollutants, especially NO_2_ which is a pollutant of particular concern in Sheffield as well as the rest of the UK. In this paper, the aim is to evaluate the monitoring capability of Envirowatch E-MOTEs for air quality monitoring. This is the first paper comparing the performance of Envirowatch E-MOTEs with each other and with reference instruments, which are recommended by the European Union and UK DEFRA for air quality monitoring. The paper analyses a year’s worth of data and provides a more detailed assessment in comparison to previous studies (which have generally analysed sensor data for a limited time ranging from a week to a couple of months). Furthermore, supervised machine learning approaches including multiple linear regression and generalised additive modelling approaches are employed to calibrate the sensors by comparing their measurements with the reference instruments and setting up the slope and intercept.

## Methodology

In this project, the aim is to analyse CO (ppm), NO and NO_2_ (ppb) data measured by LCS (Envirowatch E-MOTEs) and NO and NO_2_ (ppb) measured by reference sensors, along with meteorological data such as wind speed, temperature and relative humidity, to assess the performance of LCS. All these data were available for the period October 2016 to September 2017. In this section, firstly we describe Envirowatch E-MOTEs, their operating principle and the air quality monitoring network in Sheffield. This is followed by a statistical analysis which includes model selection, development and assessment.

### Envirowatch E-MOTEs

In this project, E-MOTEs developed by Envirowatch Newcastle, UK, were employed. The E-MOTE was launched by Envirowatch in 2010. Precision or reference instruments used for air quality monitoring are large and expensive to both purchase and maintain; in contrast, these sensors are cheaper, small and suitable for a high-density air quality monitoring network. E-MOTEs work on a similar principle as the AQMesh pods, which use the latest generation of electrochemical sensors made by alphasense. E-MOTEs were used to measure the levels of three gaseous pollutants: carbon monoxide (CO), nitric oxide (NO) and nitrogen dioxide (NO_2_).

**Fig. 1:**
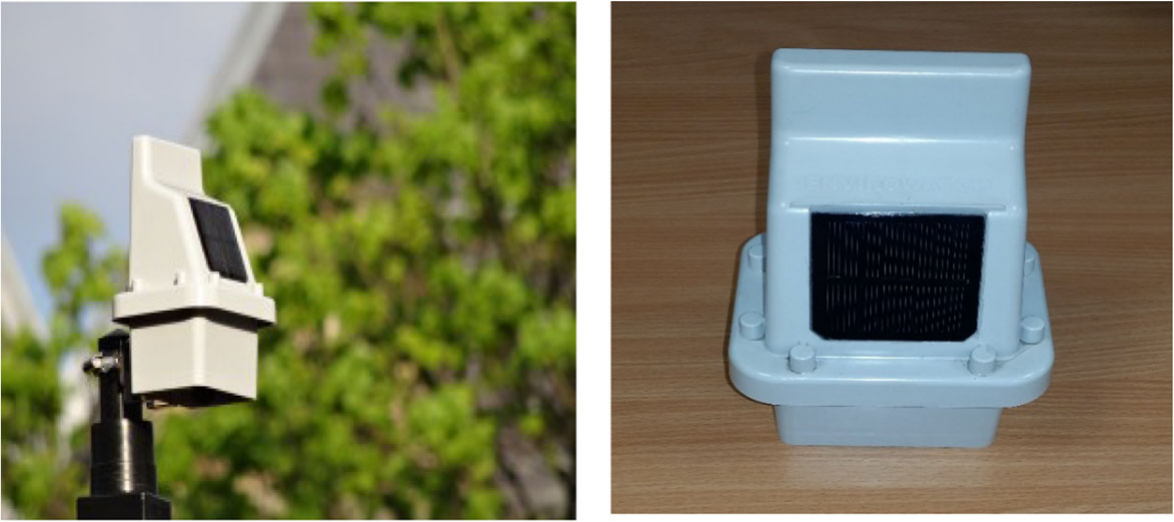
Envirowatch E-MOTEs post-mounted (left) and showing the solar panel used for battery charging (right). Ten of these E-MOTEs were used for collecting data used in this study

The E-MOTEs use wireless technology to communicate their sensor reading and can be deployed on lamp posts or other street furniture (Fig. 1). E-MOTEs in a cluster communicate with a gateway by means of the Zigbee protocol within a specific area for high-resolution monitoring. The use of this protocol allows the individual units to communicate with each other and pass data from sensors that are not in range or without line-of-sight of the gateway. Using GPRS, the gateway device communicates the collected data over an internet connection to a cloud server operated by Envirowatch. The data are post-processed and presented for access by users via the Enviroview web interface as well made available for download via an application programming interface (API).

LCS are more compact, portable and use less power as compared to reference instruments. E-MOTEs use electrochemical technology for measuring gaseous air pollutants, including NOx, CO and O_3_. Electrochemical sensors work by reacting to the target gas, generating an electrical output which varies with the concentration of target gases present in air. Independent Envirowatch E-MOTEs transmit raw measurement data to a cloud server. These data are not concentration readings as such and require post-processing. Once readings are received, mathematical processing is applied to correct cross-gas effects and prevailing environmental factors.

An electrochemical sensor contains a cell where three electrodes are present. These electrodes are known as the working or sensing electrode, counter electrode and reference electrode. The electrodes are separated by wetting filters, which are hydrophobic separators enabling ionic (cation and anion) contact between the electrodes, allowing transport of the electrolyte via capillary action. The sensed gas is either reduced or oxidised at the working electrode. These reactions are catalysed by the electrode materials specifically developed for the gas in question. Normally, the rate of diffusion of the sensed gas to the sensor electrode is slower than the rate of reaction of the gas at the electrode. Therefore, the concentration of the sensed gas determines the electrical current output by the sensor (Mead et al. 2013). The potential difference between the working and counter electrodes then generates an electric current which is the output signal of the sensor. With a resistor connected across the electrodes, a current proportional to the gas concentration flows between the anode and the cathode. Thus, the current can be measured to determine the gas concentration. The current generated by these types of electrochemical sensors is measured using suitable electronics and, following further processing, displayed as a concentration measurement in ppm (for CO) or ppb (for NOx, and O_3_).

### Air quality monitoring network (AQMN)

Air quality data analysed in this paper are mainly from two sources: LCS and reference instruments, which are described below:

1. LCS network

**Fig. 2:**
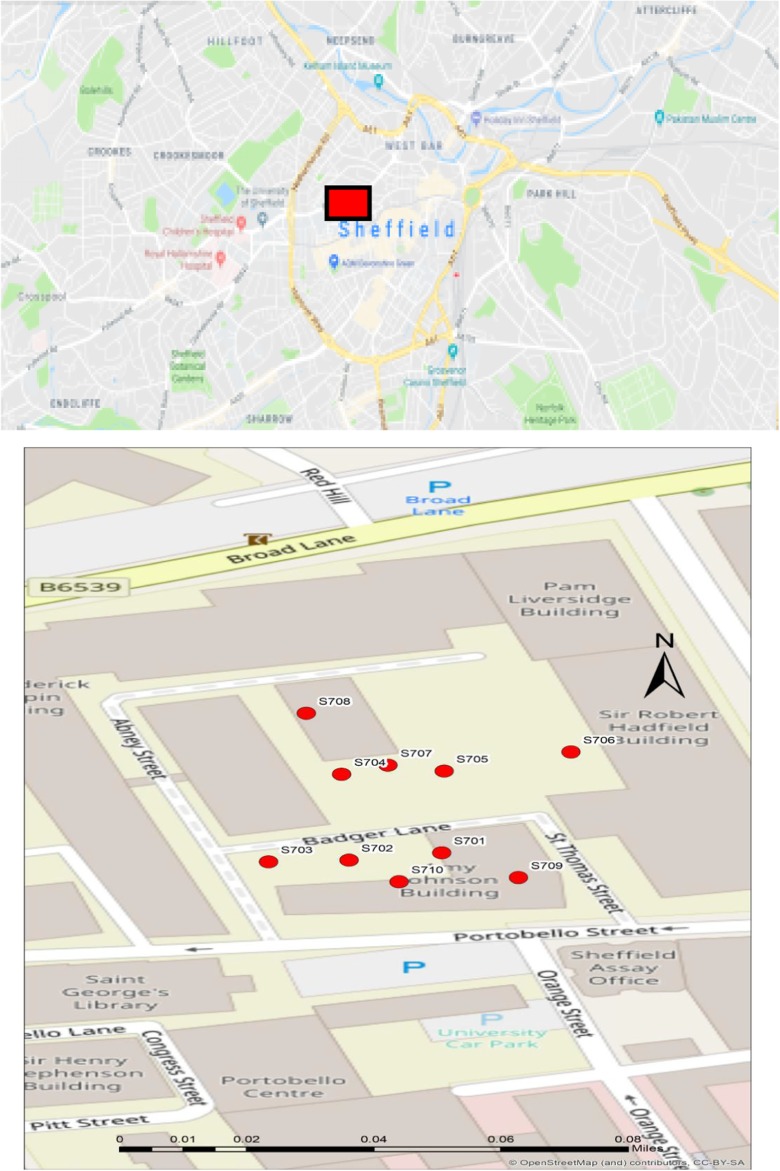
Map of the locations of the Envirowatch E-MOTEs included in this study, where the red rectangle in the upper panel shows the location where sensors were deployed and the lower panel shows their localisation sites (the map was developed in ArcMap10.4.1 using basemaps of OpenStreetMap)

LCS used for air quality monitoring were Envirowatch E-MOTEs. Ten E-MOTEs were deployed at the University of Sheffield Campus (Fig. 2) for a year (October 2016 to September 2017). This area is bounded by Mappin Street, Rockingham Street, Portobello Street and Broad Lane and can be classified as urban background area. This area is part of the University of Sheffield and is mainly comprised of offices, lecture theatres and student accommodation. E-MOTEs provide minute-by-minute air pollutant measurements, which were converted to hourly averages to make them comparable to the data collected by reference instruments. Sensor identities and coordinates of their locations are shown in Table 1 along with the average annual concentration of each pollutant measured.

(2) Reference instruments network

**Table 1:** Coordinates of the sensors and data summary showing the mean concentrations (annual mean) of various air pollutants from October 2016 to September 2017

Sensors ID	Northing (m)	Easting (m)	CO (ppm)	NO (ppb)	NO_2_ (ppb)
S701	392,846	631,411	0.33	2.39	58.00
S702	392,846	631,425	0.46	20.31	16.60
S703	392,845	631,437	0.33	10.95	13.30
S704	392,878	631,425	0.33	3.70	18.85
S705	392,878	631,409	0.35	11.55	19.03
S706	392,883	631,390	0.43	15.98	18.60
S707	392,878	631,418	0.33	7.87	17.59
S708	392,900	631,429	0.33	9.60	17.93
S709	392,837	631,400	0.33	9.49	18.70
S710	392,837	631,418	0.32	5.66	17.07

**Fig. 3:**
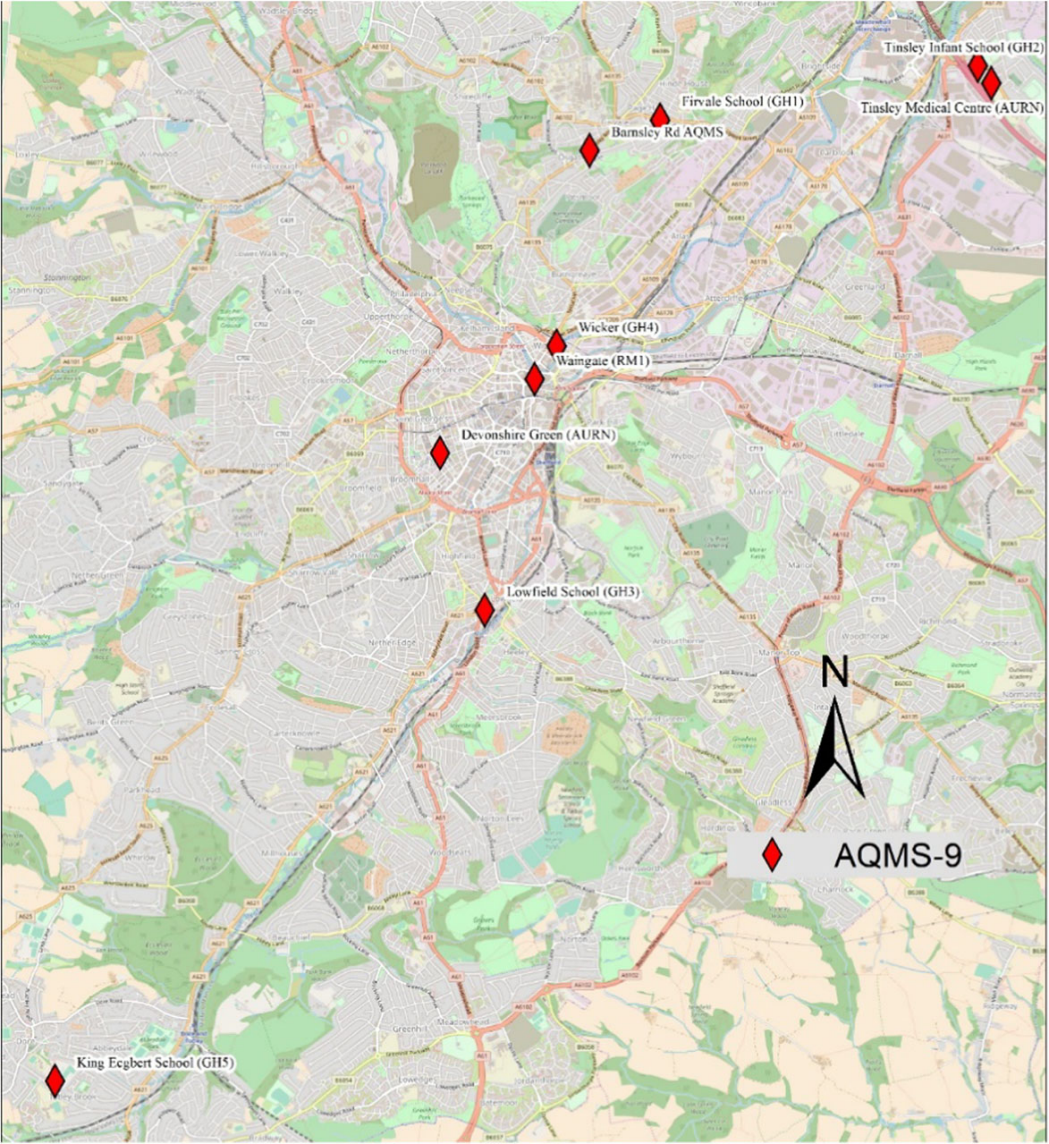
Air quality monitoring network of continuous monitoring stations in Sheffield comprised of AURN sites run by DEFRA and Sheffield City Council sites (the map was developed in ArcMap10.4.1 using basemap of OpenStreetMap)

**Fig. 4:**
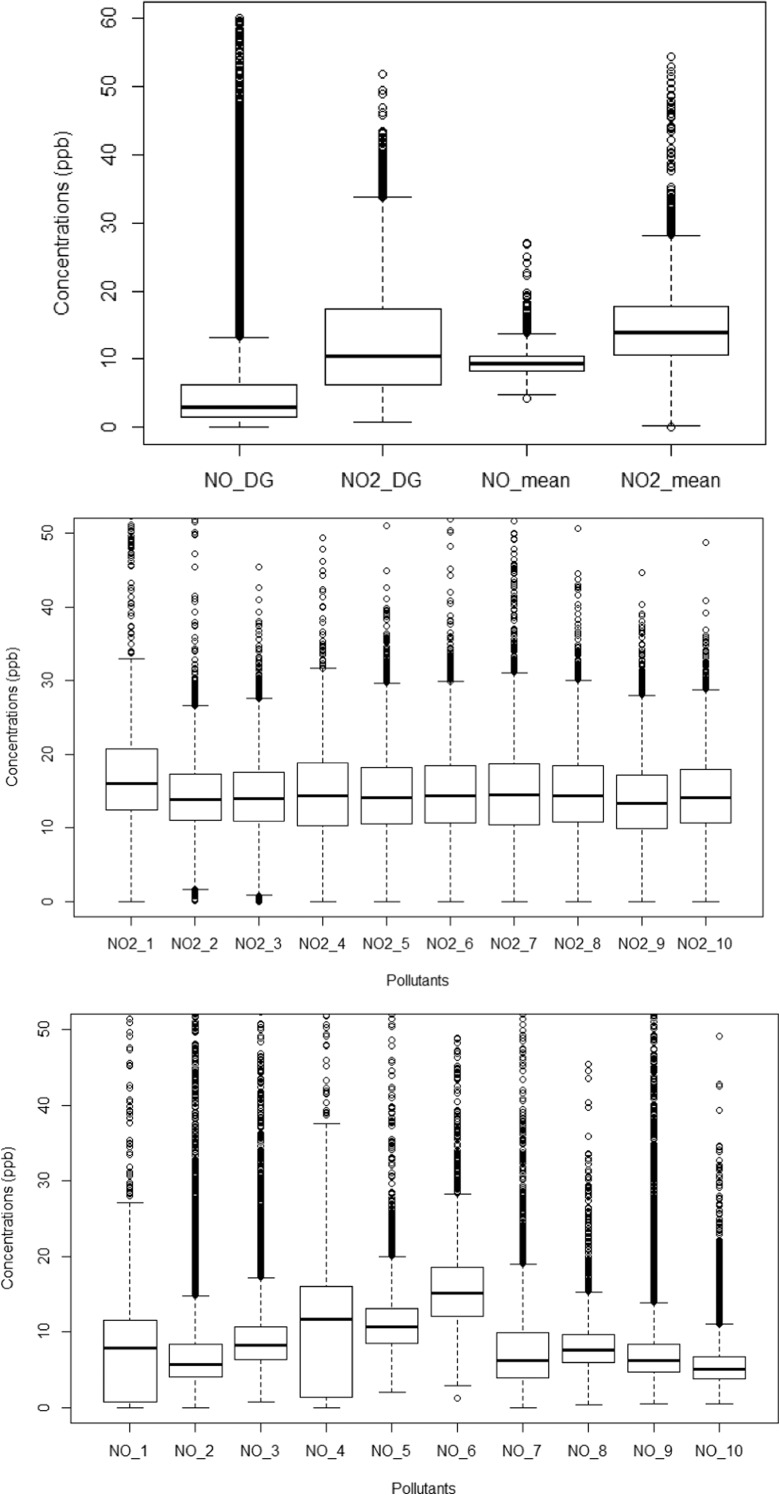
Box plots of hourly concentrations (ppb) NO (lower panel), NO_2_ (centre panel) measured by E-MOTEs and their mean compared with reference measurements from Devonshire Green monitoring station (upper panel)

Several reference instruments are installed to monitor various air pollutant concentrations in Sheffield. These total nine (9) continuous air quality monitoring stations (AQMS) and provide hourly concentrations of air pollutants, including NOx, CO, SO_2_, O_3_ and particulate matter mainly PM_10_ and PM_2.5_. Out of these, three (3) of the monitoring stations are part of the Automatic Urban and Rural Network (AURN) run by the UK government’s DEFRA, whereas the remaining six sites are installed and managed by Sheffield City Council (Fig. 3). Devonshire Green (AURN), Waingate (RM1) and Wicker (GH4) are the nearest to the E-MOTE network. However, data from October 2016 to September 2017 were available only from Devonshire Green (DG) monitoring station, which are compared with data from the installed sensors. Figure 4 shows box plots comparing NO (lower panel) and NO_2_ concentrations (middle-panel) measured by each of the E-MOTEs and with reference sensors (upper panel). The box plots show the distribution of the concentrations with some descriptive statistics including median (middle line of the box), lower or first quartile (lower end of the box), upper or third quartile (upper end of the box), inter-quartile range (representing middle 50% of the data points), upper and lower whiskers representing concentrations outside the middle 50% and outliers (point lying beyond the whiskers). Box plots compare both central tendency and variability or distribution of the concentrations. NO_2_ concentrations measured by the various sensors exhibit a similar pattern; in contrast, NO concentrations show much more variability.

### Statistical analysis

Statistical analyses were carried out, comprising correlation analysis, regression analysis and graphical presentations, in the base packages of the R programming language (R Core Team 2017) and two of its additional packages known as ‘openair’ (Carslaw 2016) and ‘mgcv’ (Wood 2017).

In this paper, supervised machine learning approaches are suggested for calibrating E-MOTE outputs in comparison with measurements gathered from the reference instruments. Although these sensors are pre-calibrated by the manufacturers, they require local outfield calibration to account for cross interference of other pollutants and meteorological parameters, e.g. temperature and relative humidity. Two modelling approaches are employed in this study: (a) linear regression models (LRM) and (b) generalised additive models (GAMs). For details on these models, see Hastie and Tibshirani (1990), Wood (2006), Munir et al. (2013) and Sayegh et al. (2014).

#### Model selection: choosing the best set of predictors

**Fig. 5:**
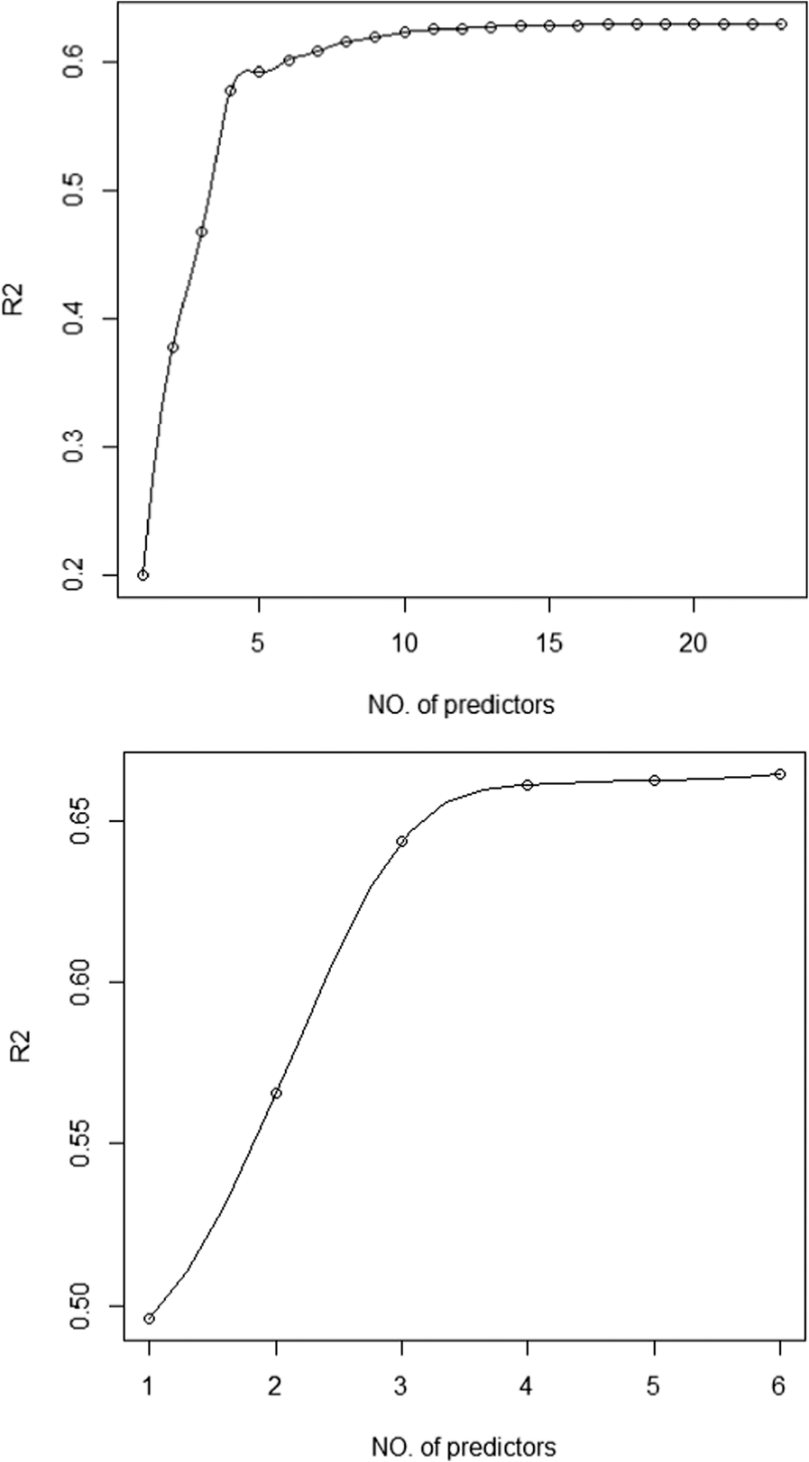
Best subset regression (BSR) using 23 predictors (NO_1 to NO_10, NO_2__1 to NO_2__10, wind speed (WS), temperature (Temp) and relative humidity (RH)) for predicting NO_2__DG (upper panel) and 6 predictors (NO_mean, NO_2__mean, NO_DG, WS, Temp and RH) for predicting NO_2__DG (lower panel)

Air pollutant data were obtained from ten E-MOTEs and a reference AQMS each measuring NO and NO_2_. Meteorological data of wind speed, relative humidity and temperature were also available from a weather station collocated with reference station. Firstly, NO and NO_2_ from all ten E-MOTEs (making 20 variables) along with relative humidity, wind speed and temperature were considered as predictors (independent variables) for predicting the concentration of NO and NO_2_ measured by the reference instrument (Fig. 5, upper panel). Various other combinations of predictors were also tested to find the best set of predictors using best subset regression (BSR). After testing a combination of various predictors, six predictors were chosen and were used in the model development to model the concentrations of NO_2_ and NO measured by reference instrument. It can be seen in (Fig. 5 upper panel) that the value of *R*^2^ increases with an increase in the number of independent variables; however, after adding a certain number of covariates, the line becomes horizontal showing little improvement in the *R*^2^ value. Considering the results of BSR and the outputs of the actual LRM and GAM (discussed in coming sections), the final number of covariates were decided. The whole dataset was divided into two subsets: a training dataset (75%) and a testing dataset (25%) both selected randomly. The raining dataset was used to train the model, whereas the testing dataset was used to assess the model’s performance and check its validity.

The model selection process examines all possible sets of predictors in ordinary least square (OLS) regressions and leads to choosing one that fits best according to some criterion. The criterion could be based on *p* value as in the standard stepwise methods (e.g. backwards stepwise regression), which take one variable away and then re-examine the model. Alternatively, the criterion could be based on *R*^2^ or adj-*R*^2^. This is called BSR or leaps-and-bounds approach. Criterion based on *R*^2^ and adj-*R*^2^ is technically much stronger than on the *p* value; therefore, in this paper, the leaps-and-bounds method is adopted. To apply the leaps-and-bounds method, we employed one of the package of R programming language known as ‘Leaps’ to select the best set of predictors.

#### Model development

In this paper, two modelling approaches are employed: linear regression model (LRM) and generalised additive model (GAM).

- LRM

Two types of linear models were developed: Simple linear regression and multiple linear regression model. In simple linear regression model, only one dependent variable (predictor) was used. This helps correct slopes and offsets (intercepts) values of the lower-cost sensors to improve the accuracy of results. During calibration, the measurements are regressed vs reference measurements, where readings from the E-MOTEs (NO_mean or NO_2__mean) are taken as independent (x-axis) and reference readings (NO_DG or NO_2__DG) as the dependent (y-axis) variable. The regression model is run and values of slopes and intercepts are calculated as shown in Eqs. 1 and 2; here, DG stands for Devonshire Green which is the location of a reference air quality monitoring station and NO_mean is the average of the readings from all the sensors.

1 $$ \mathrm{NO}\_\mathrm{DG}={\upbeta}_{\mathrm{o}}+{\upbeta}_1\left(\mathrm{NO}\_\mathrm{mean}\right)+\upvarepsilon $$

2 $$ {\mathrm{NO}}_2\_\mathrm{DG}={\upbeta}_{\mathrm{o}}+{\upbeta}_1\left({\mathrm{NO}}_2\_\mathrm{mean}\right)+\upvarepsilon $$

The values of slopes and intercepts are then applied to the whole dataset of E-MOTEs.

β_o_ is the intercept, β1 is the coefficient or slope, Ɛ is the error term (the difference between observed and modelled concentrations).

To account for cross interference and for the effect of meteorological parameters, a multiple linear regression model was developed for each NO and NO_2_ value as given in Eqs. 3 and 4 using the predictors selected in the model selection section (3.2.1).

3 $$ \mathrm{NO}\_\mathrm{DG}={\upbeta}_{\mathrm{o}}+{\upbeta}_1\ \left(\mathrm{NO}\_\mathrm{mean}\right)+{\upbeta}_2\ \left({\mathrm{NO}}_2\_\mathrm{DG}\right)+{\upbeta}_3\ \left({\mathrm{NO}}_2\_\mathrm{mean}\right)+{\upbeta}_4\ \left(\mathrm{WS}\right)+{\upbeta}_5\ \left(\mathrm{RH}\right)+{\upbeta}_6\ \left(\mathrm{Temp}\right)+\upvarepsilon $$

4 $$ {\mathrm{NO}}_2\_\mathrm{DG}={\upbeta}_{\mathrm{o}}+{\upbeta}_1\ \left(\mathrm{NO}\_\mathrm{DG}\right)+{\upbeta}_2\ \left({\mathrm{NO}}_2\_\mathrm{mean}\right)+{\upbeta}_3\ \left(\mathrm{NO}\_\mathrm{mean}\right)+{\upbeta}_4\ \left(\mathrm{WS}\right)+{\upbeta}_5\ \left(\mathrm{RH}\right)+{\upbeta}_6\ \left(\mathrm{Temp}\right)+\upvarepsilon $$

In the above equations, β_o_ is the intercept, β1 to β6 are the coefficients or slopes and Ɛ is the error term. Furthermore, NO_mean and NO_2__mean are average concentrations of NO and NO_2_ from the lower-cost sensors, NO_DG and NO_2__DG are NOx concentrations from the Devonshire Green monitoring station, WS is wind speed (m/s), RH is relative humidity (%) and Temp is the air temperature (°C).

(b) GAMs

GAMs are advanced modelling techniques which are applicable to both normal and non-normal data distribution and do not assume the relationship between response and explanatory variables to be linear. GAMs rather permit the response probability distribution to be any member of the exponential family (e.g. normal, exponential, gamma and poisson distribution). In contrast, a linear model assumes the response distribution to be normal and the relationship between response and explanatory variables to be linear.

The GAM models developed in this study are shown in Eqs. 5 to 8 below, using the same predictors used by LRM shown in Eqs. 1 to 4.

5 $$ \mathrm{NO}\_\mathrm{DG}=\mathrm{s}1\ \left(\mathrm{NO}\_\mathrm{mean}\right)+\upvarepsilon $$

6 $$ {\mathrm{NO}}_2\_\mathrm{DG}=\mathrm{s}1\ \left({\mathrm{NO}}_2\_\mathrm{mean}\ \right)+\upvarepsilon $$

7 $$ \mathrm{NO}\_\mathrm{DG}=\mathrm{s}1\ \left(\mathrm{NO}\_\mathrm{mean}\right)+\mathrm{s}2\ \left({\mathrm{NO}}_2\_\mathrm{DG}\right)+\mathrm{s}3\ \left({\mathrm{NO}}_2\_\mathrm{mean}\right)+\mathrm{s}4\ \left(\mathrm{WS}\right)+\mathrm{s}5\ \left(\mathrm{RH}\right)+\mathrm{s}6\ \left(\mathrm{Temp}\right)+\upvarepsilon $$

8 $$ {\mathrm{NO}}_2\_\mathrm{DG}=\mathrm{s}1\ \left(\mathrm{NO}\_\mathrm{DG}\right)+\mathrm{s}2\ \left({\mathrm{NO}}_2\_\mathrm{mean}\right)+\mathrm{s}3\ \left(\mathrm{NO}\_\mathrm{mean}\right)+\mathrm{s}4\ \left(\mathrm{WS}\right)+\mathrm{s}5\ \left(\mathrm{RH}\right)+\mathrm{s}6\ \left(\mathrm{Temp}\right)+\upvarepsilon $$

In the above models (5 to 8), s1 to s6 are the smoothing terms (Wood 2006), each one of these is associated with the adjacent explanatory variable. Response or modelled variables are given on the left and the explanatory variables of each model are given on the right of the equations.

#### Models’ assessment

To evaluate the models’ performance, predicted and measured (observed) concentrations were compared. For this purpose, several statistical metrics were calculated including correlation coefficient (*r*), coefficient of determination (*R*^2^), root mean square error (RMSE), normalised mean biased (NMB), factor of two (FAC2) and coefficient of efficiency (COE), which are defined by Carslaw (2016) and Sayegh et al. (2014). RMSE provides a good measure of the model error by calculating how close or far the predicted values are to the observed values. NMB estimates average over or under prediction, whereas ‘r’ is the strength of the linear relationship between two variables (here, modelled and observed concentrations). NMB value between + 0.02 and − 0.02 shows acceptable model performance. We would like ‘r’ to have a value as close to one (± 1) as possible; however, generally, a value ranging from ± 0.5 to ± 0.99 indicates reasonably good performance. FAC2 is the fraction of modelled values within a factor of 2 of the observed values. FAC2 should satisfy the condition that 0.5 ≤ Mi/Oi ≤ 2, where Mi represents the modelled values and Oi represents the observed values. A highly efficient or perfect model should have COE value of 1; however, when analysing real data, a model should have a COE value of less than 1. COE having a zero value (COE = 0) means the model prediction is not better than the mean of the observed value, which in other words means its prediction power is zero; it has no predictive advantage.

## Results and discussion

### Temporal variability and correlation analysis

Hourly average NO_2_ (ppb), NO (ppb) and CO concentrations (ppm) measured by ten E-MOTEs seemed reasonable and had an overall mean of about 22 ppb, 10 ppb and 0.35 ppm, respectively. Overall, various air pollutant concentrations showed a similar pattern at different monitoring sites during different seasons, for instance, NO_2_ concentration was higher in winter months and lower in summer (time plots not shown for brevity). These seasonal trends are further analysed in coming sections. NO_2_ and NO concentrations measured at the Devonshire Green monitoring site also showed higher concentrations in colder months and lower concentrations in warmer months. Obara et al. (2011) and Cai et al. (2016) have reported that air pollutant levels are strongly associated with stable weather conditions, atmospheric inversion, low wind speed and shallow boundary layer which are generally found in winter seasons in the UK. In such meteorological conditions, air pollutants emitted by various sources do not disperse and stay near the emission sources due to poor horizontal and vertical dispersion.

**Fig. 6:**
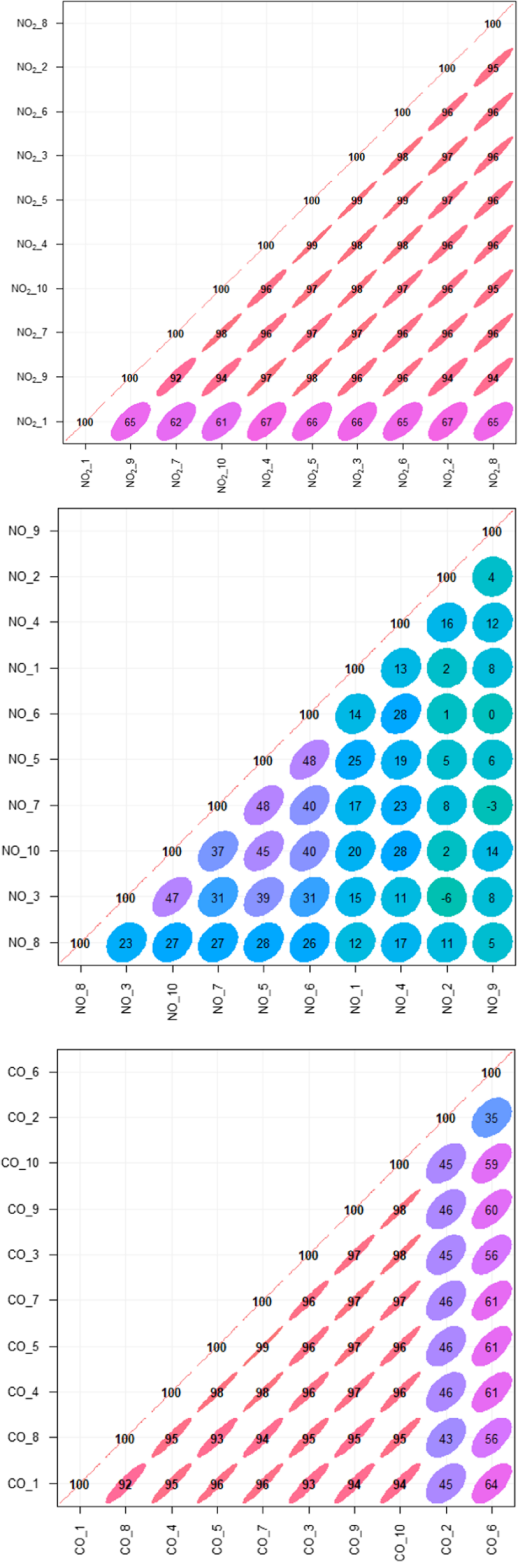
Correlation plots of NO_2_ ppb (upper panel), NO ppb (centre panel) and CO concentrations (ppm) (lower panel) from ten E-MOTEs during Oct 2016 to Sept 2017 in Sheffield. All *r* values should have been presented as decimal number; however, here, both zero and decimal points are avoided to facilitate presentation

Figure 6 shows correlation plots of hourly average NO_2_ (upper panel), NO (centre panel) and CO (lower panel) concentrations collected by the ten E-MOTEs. The correlation coefficient value, ranging from − 1 to + 1, are normally represented as a decimal number (e.g. 0.xx). However, here to facilitate presentation, both zero and decimal points are avoided, following the default format of ‘openair’ suggested by Carslaw (2016). NO_2_ concentrations show very strong positive correlation between various sensors. Mostly, correlation coefficients are greater than 0.92 (*r* > 0.92), except sensor-1 (NO_2__1), which shows relatively weaker correlation, with *r* values ranging from 0.60 to 0.67. The cause of this weaker correlation is likely due to erroneous data caused by bad communication between the sensor and the gateway. Taking this into account, this shows all the E-MOTE measurements of NO_2_ are consistent with each other and show strong similarity with each other. This strong similarity puts confidence in the consistency of these sensors. This is the first study reporting the performance of E-MOTEs; therefore, no comparison was possible with previous studies. However, several researchers have assessed the performance of other LCS, such as AQMesh pods both in the UK and Europe and reported that their performance varied both spatially and temporally from sensor to sensor (Castell et al. 2017).

In contrast, NO concentrations (Fig. 6, middle-panel) showed weaker correlation. NO_5 vs NO_6 and NO_5 vs NO_7 showed strongest correlation with *r* value of 0.48 each. NO_6 vs NO_9 show zero *r* value, whereas NO_2 vs NO_3 showed negative correlation. Figure 6 (lower panel) presents correlation plots of CO concentrations showing much stronger correlation than NO concentrations. Except for CO_2 and CO_6, the remaining sensors compared against each other showed *r* values greater than 0.90. CO_2 and CO_6 have *r* values ranging from 0.35 to 0.64, which are those for CO_2 vs CO_6 and CO_1 vs CO_6, respectively. This confirms that E-MOTEs produce consistent measurements of CO concentration. For further analysis, time variation plots are constructed in the next section to see how the pollutant concentrations vary at various time scales, such as diurnal, weekly and annually.

**Fig. 7:**
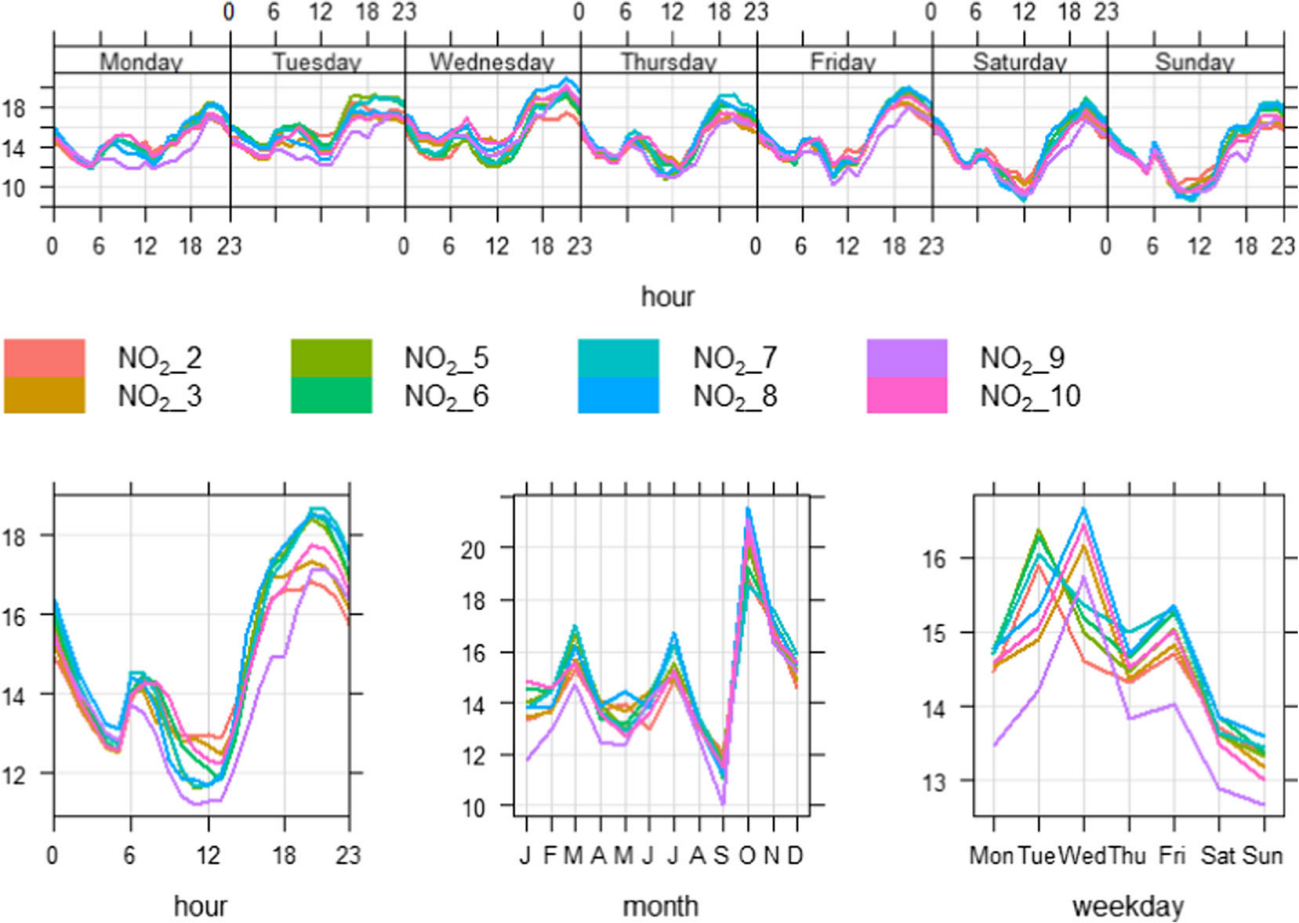
Time variation plots of NO_2_ concentrations (ppb) from nine sensors from October 2016 to September 2017 (readings from one sensor, NO_2__1 were excluded due to missing and erroneous data)

**Fig. 8:**
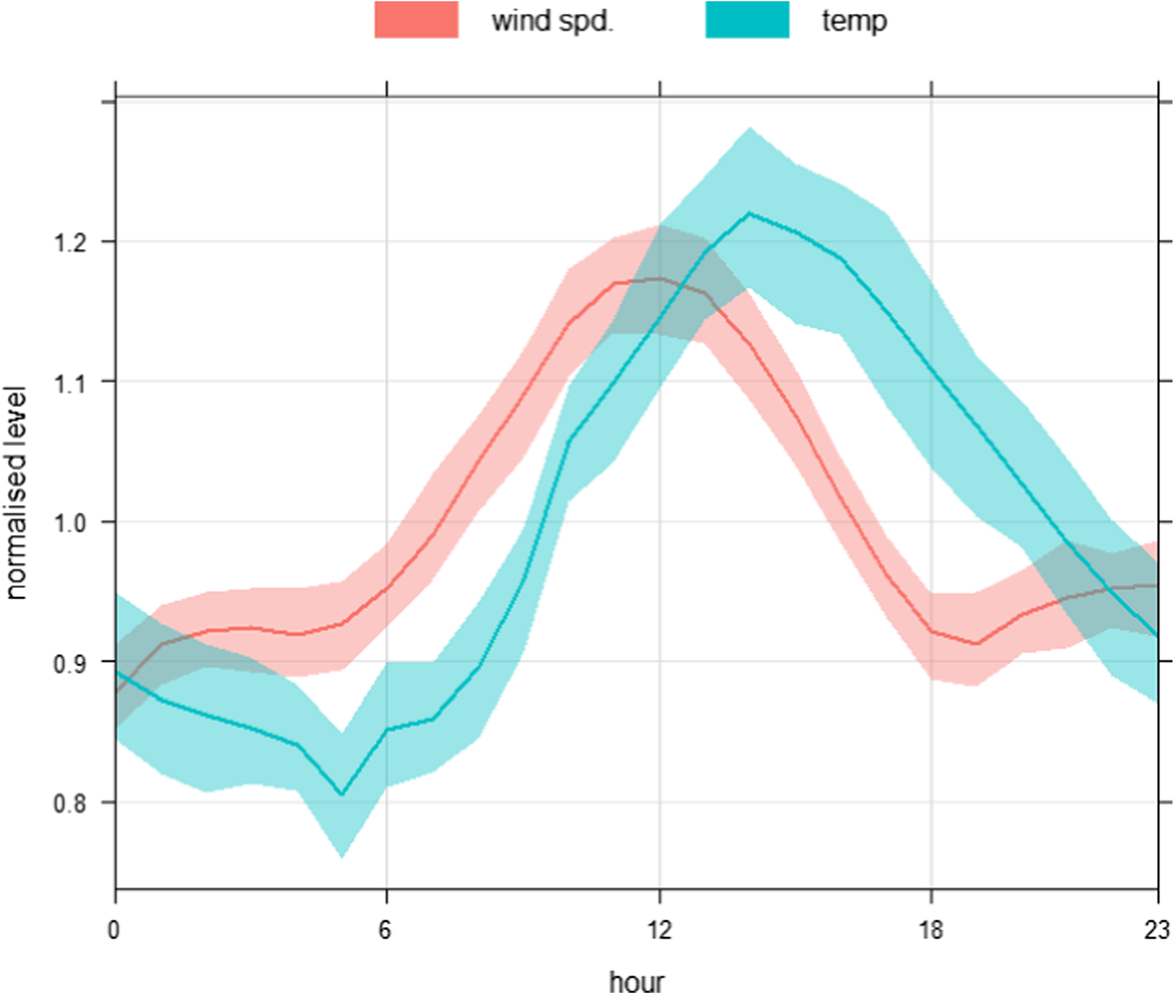
Diurnal cycles of wind speed (m/s) and temperature (°C) at the Devonshire Green monitoring station during 2017, showing highest wind speed and temperature during the afternoon

Figure 7 shows time variation plots of NO_2_ concentrations (ppb) collected by nine of the E-MOTEs. NO_2__1 was removed due to missing and likely incorrect measurements. These plots show strong similarities among the nine sensors on all time scales, i.e. diurnal, weekly and annual cycles. During the diurnal cycle (Fig. 7, lower-left-panel), NO_2_ concentrations (ppb) start decreasing after midnight and continue to do so until about 05:00 h, then slightly increase at about 06:00–08:00 h probably due to morning traffic peak hours. Afterwards, NO_2_ levels gradually decrease and reach a minimum level around midday (12:00 h), most probably due to low traffic activities and atmospheric conditions which help disperse air pollutants quickly. Relatively high temperature, high wind speed and wider atmospheric boundary layer during the afternoon improve both horizontal and vertical air pollutant dispersion. Diurnal cycles of temperature (°C) and wind speed (m/s) during 2017 at the Devonshire Green monitoring stations are shown in Fig. 8, which clearly shows that wind speed and temperature reach the highest levels during the afternoon, which leads to a widening of the atmospheric boundary layer and help disperse locally emitted pollutants. After 14:00 h, NO_2_ levels begin increasing and reach their highest levels in response to the evening’s busiest traffic hours (about 18:00–20:00 h), when this activity cause pollutant emissions to increase. Furthermore, in the evening, the atmosphere is colder and more stable which discourages air pollutants dispersion. The stable atmosphere continues as the night progresses, although traffic levels decline. This reduction in traffic levels results in a slight decrease in NO_2_ levels. It is worth noting that all the sensors produce almost the same temporal pattern on daily basis. Diurnal cycles on individual days (Monday to Sunday) are shown in Fig. 7 (upper panel). Weekly cycles of NO_2_ concentrations (ppb) are shown in Fig. 7 (lower-right-panel), where a uniform pattern of various sensors can be observed. As expected, different traffic patterns during the weekend result in lower levels of NO_2_ on Saturday and Sunday.

**Fig. 9:**
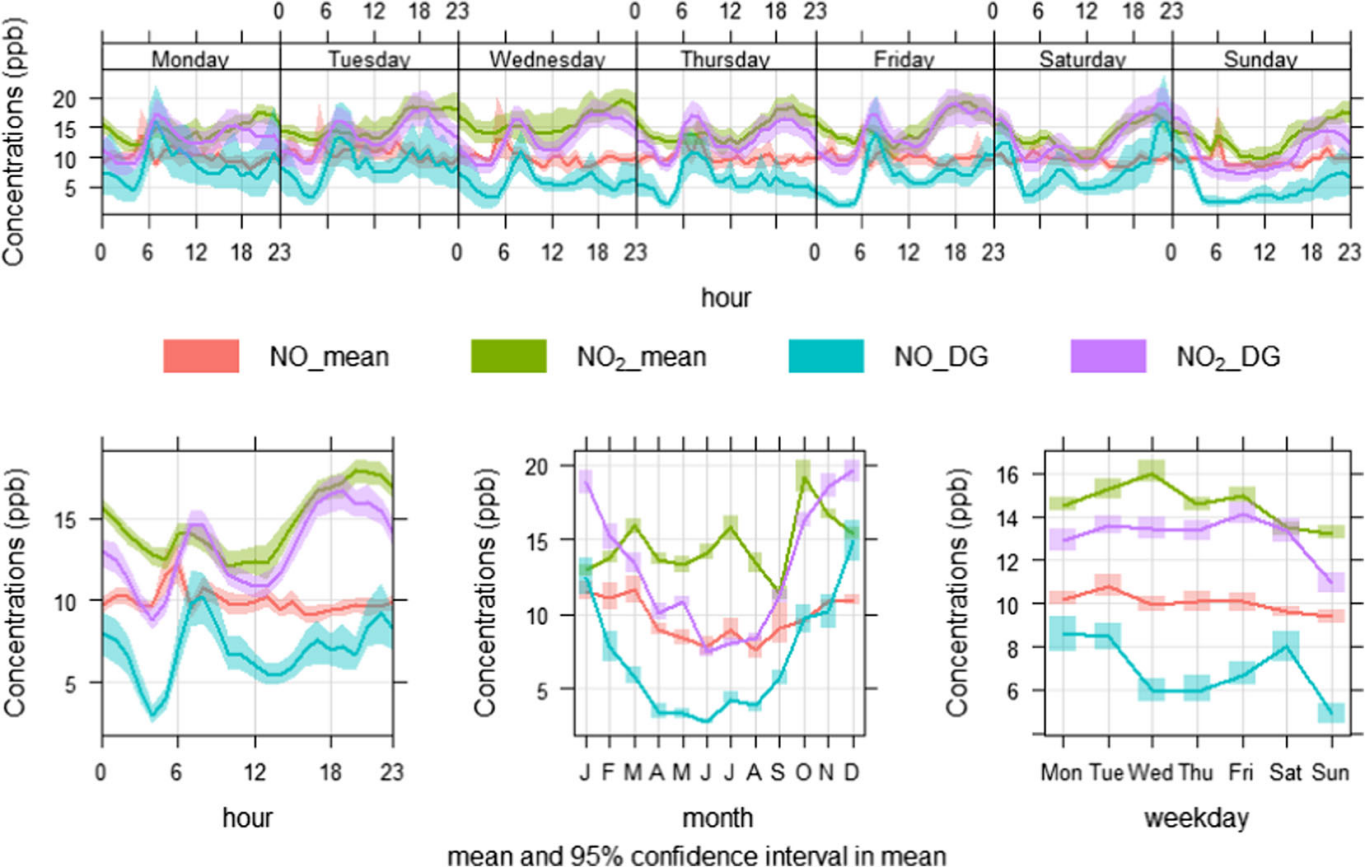
Time variation plots comparing diurnal, weekly and annual cycles of NO_2_ and NO at Devonshire Green and the mean of all 10 E-MOTE sensors during Oct 2016 to Sept 2017 in Sheffield

Annual cycles of NO_2_ (Fig. 7, lower-middle-panel) are somewhat confusing showing much higher levels of NO_2_ during October. It was expected that NO_2_ levels would have been higher during the colder months (i.e. November, December and January) and lower during the hotter months (i.e. May, June and July). This is seen in Fig. 9, which depicts NO_2_ levels measured at the Devonshire Green monitoring station during the same period as shown in Fig. 7. Concentrations measured at this location are shown as NO_DG and NO_2__DG, and average concentrations of the E-MOTEs are shown as NO_mean and NO_2__mean. CO is not monitored at this site and therefore comparison with the E-MOTEs was not possible. All E-MOTE sensors have a strong correlation with each other and have the same temporal pattern; therefore, it is convenient to average their measurements to facilitate comparison with the measurements from the Devonshire Green site. NO_2__mean and NO_mean are closely related with NO_2__DG and NO_DG at diurnal, weekly and annual cycles; however, some differences can be observed at various temporal intervals. To summarise, it can be said that generally, E-MOTEs show close similarities with the reference instrument; however, there are some dissimilarities at various temporal scales. NO_2_ and NO concentrations (ppb) at Devonshire Green produced a smooth annual cycle going down from January to June–July and then going up until December. Such a smooth annual cycle does not exist when mean NO and NO_2_ concentrations measured by E-MOTEs were plotted. NO_2__mean showed lowest level in September and highest in October and the clear summer and winter difference demonstrated by Devonshire Green has disappeared here. Overall, the results discussed above are encouraging as they successfully capture the temporal trends of air pollutants and show a consistent performance by showing strong correlation with each other.

### Modelling

In this section, both linear and non-linear regression modelling approaches are employed and their performances are compared using several statistical metrics.

#### Linear regression models

**Fig. 10:**
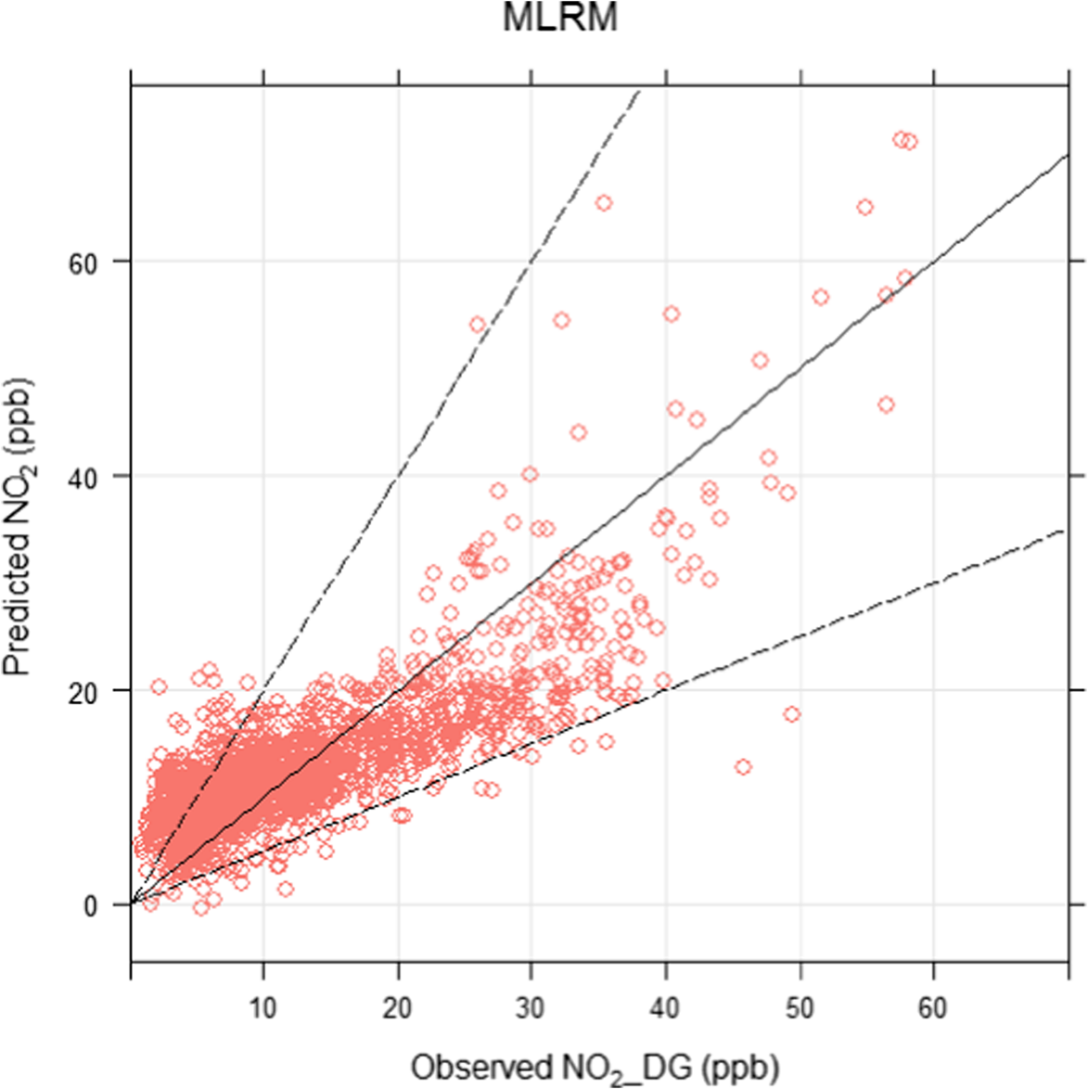
Scatter plot comparing observed and MLRM-predicted concentrations of NO_2__DG (ppb) based on the testing data (25% randomly selected), where the solid middle line is the 1:1 line, whereas the upper and lower lines represent 2:1 and 0.5:1 respectively. Most of the points lie within these lines demonstrating acceptable model performance

The outputs of model 1 to 4 are presented in Table 2, showing the values of various statistical metrics. Table 2 shows that the multiple linear regression model (MLRM) demonstrated much better performance than the simple linear regression model (SLRM). This was expected as MLRMs used several extra explanatory variables including temperature, wind speed and relative humidity. The values of FAC2, RMSE, *R*^2^, NMB and COE are shown in Table 2. The values of NMB demonstrate acceptable model performance since they lie within the range of + 0.02 to − 0.02 (Table 2). The other metrics also signify a small degree of error in the model and good predictability. Figure 10 shows a scatter plot with model lines and shows that most of the points lie between the FAC2 region, which again demonstrates acceptable model performance. It should be noted that these metrics were calculated using the testing data (25% randomly selected), and for the training dataset, the values returned for these metrics displayed even better performance (not shown for brevity). This shows that using air quality data measured by LCS and meteorological data as explanatory variables, we can successfully predict (reproduce) NO_2_ concentrations measured by reference instruments. Further details of model 4 are given in Table 3, which shows that all explanatory parameters in the model had highly significant effects (*p* value < 0.01) on the response variable. Explanatory variables with positive coefficients (i.e. NO_mean and NO_2__mean) show positive effect on the response variable, whereas the variables with negative coefficients (e.g. temperature and wind speed) show negative effect on the response variable. The negative effect of temperature and wind speed suggests that warmer and windier conditions help disperse locally emitted pollutants and hence decrease NO_2_ concentrations. The negative correlation between relative humidity and temperature is well known; therefore, relative humidity is showing positive associations with NO_2_. Positive association between different NOx species is expected as they have the same emission source and therefore show positive coefficients in Table 3. Linear regression is unable to address the non-linear relationship between response and explanatory variables; therefore, a non-linear regression model is employed in the next section to test how it performs in comparison to its linear counterpart.

**Table 2:** Showing the outputs of simple (SLRM) and multiple linear regression models (MLRM)

Model	Response variable	Explanatory variable(s)	FAC2	RMSE	*R* ^2^	NMB	COE
SLRM	NO_DG	NO_mean	0.98	2.84	0.25	0.002	0.10
SLRM	NO_2__DG	NO_2__mean	0.78	10.15	0.15	0.013	0.05
MLRM	NO_DG	NO_mean, WS, NO_2__mean, RH, NO_2__DG, Temp	0.30	12.79	0.51	0.012	0.12
MLRM	NO_2__DG	NO_mean, WS, NO_2__mean, Temp NO_DG, RH,	0.83	5.76	0.64	0.001	0.41

**Table 3:** Showing various parameters of model 4 along with their slopes and *p* values

Explanatory Variable	Coefficient (slopes)	Significance value (*p* value)
Intercept	14.74	0.000***
NO_mean	0.125	0.000 ***
NO_DG	0.250	0.000***
NO2_mean	0.168	0.000***
Temp	− 0.412	0.000***
WS	− 1.219	0.000***
RH	0.026	0.001 **

Note: *p*. stars relate to how statistically significant the effect is: *p* < 0.001 = ∗∗∗, *p* < 0.01 = ∗∗

#### Generalised additive model

**Fig. 11:**
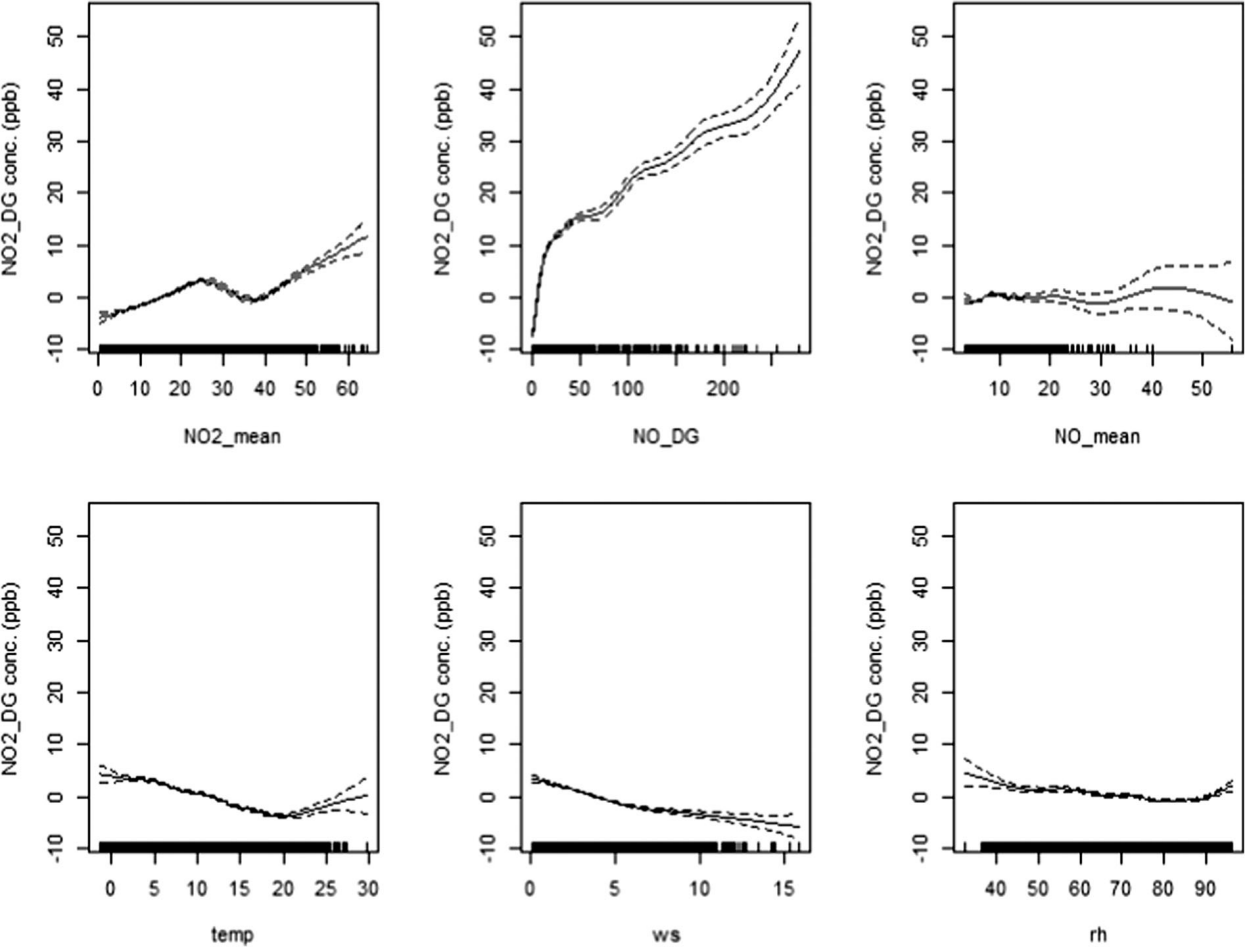
Outputs of GAM (Eq. 8), in which NO_2__DG (ppb) was used as the response variable and NO_2__mean (ppb), NO_DG (ppb), NO_mean (ppb), temperature (temp °C), wind speed (ws m/s) and relative humidity (rh %) were used as explanatory variables. The dashed lines are the estimated 95% confidence interval, whereas the vertical short lines on the x-axis show the data presence

**Fig. 12:**
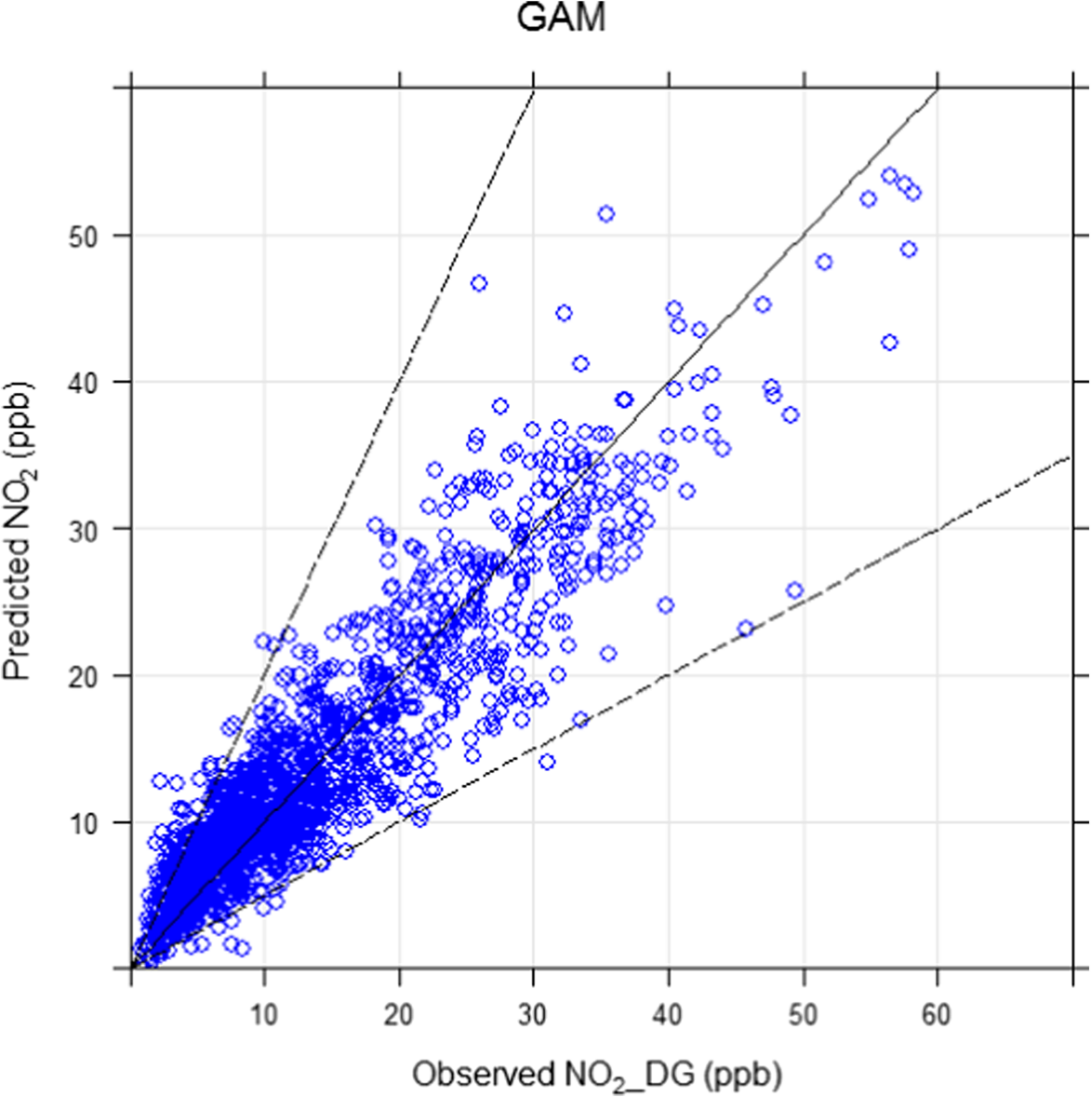
Scatter plot comparing observed and GAM-predicted concentrations of NO_2__DG (ppb) based on the testing data (25% randomly selected), where the solid middle line is the 1:1 line, whereas the upper and lower lines are 2:1 and 0.5:1 lines respectively. The dashed lines show within the factor of two regions. Most of the points lie within these lines showing an acceptable model performance

**Fig. 13:**
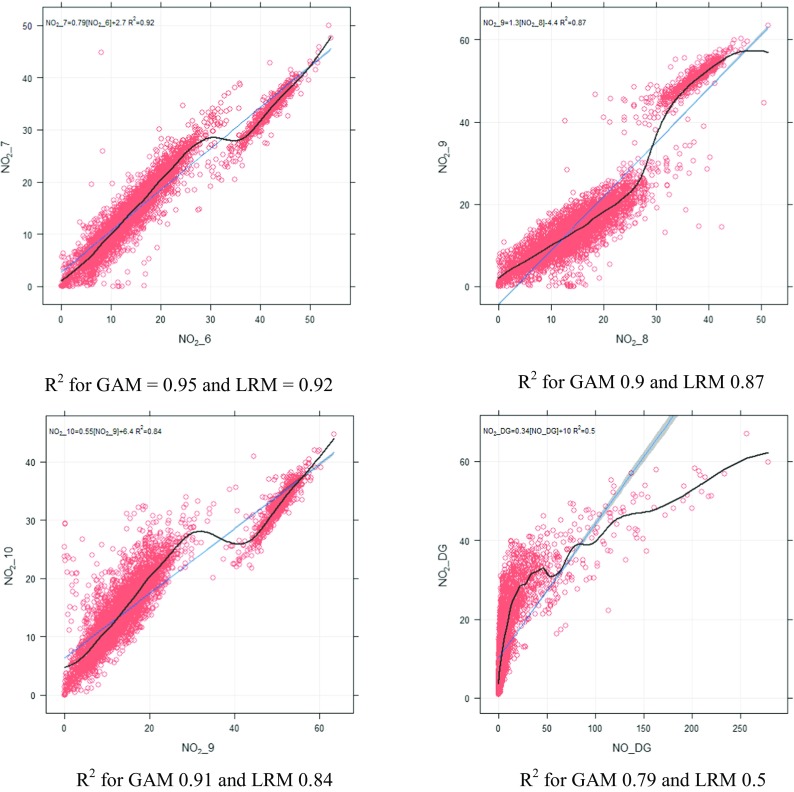
Comparing the performance of linear (LRM) and non-linear (GAM) models. *R*^2^ for GAM = 0.95 and LRM = 0.92, *R*^2^ for GAM 0.9 and LRM 0.87, *R*^2^ for GAM 0.91 and LRM 0.84, *R*^2^ for GAM 0.79 and LRM 0.5

Generalised additive models (GAM) are shown in Eqs. 5 to 8. After running these models, predicted and observed concentrations were compared and several metrics were calculated to assess their performance, which are presented in Table 4. Comparing Tables 2 and 4, it can be observed that using the same explanatory variables, GAM performs better and displays greater predictability. Comparing these models, model 8 showed best performance. Its outputs are shown in Fig. 11, which shows how the response variable (NO_2__DG) changes with each explanatory variable. This figure also shows that the association between explanatory variables and response variable (NO_2__DG) is not linear and changes for different values of the explanatory variables. It is interesting to see that the effect of temperature on NO_2_ is negative (the curve is downward) until around 20 °C is reached; afterwards, as temperature increases further, the curve turns upward, showing a positive effect, most probably due to the formation of secondary NO_2_ in the atmosphere. In contrast, the effect of wind speed results in a downward curve regardless of wind speed, which is probably due to the fact that high wind speed disperses locally emitted pollutants more effectively. GAM successfully address the non-linear relationship between response and explanatory variables, and probably, this is the reason that GAM performs significantly better than the MLRM, using the same explanatory variables. As an example, let us compare the GAM and MLRM based on NO_2__DG. GAM has resulted in a high *R*^2^ value (0.83) and lower RMSE (3.91) than MLRM where the *R*^2^ value was 0.64 and RMSE was 5.76. This shows that GAM has predicted NO_2__DG more accurately. Figure 12 compares observed and predicted NO_2_ and the plot shows a linear association between observed and predicted concentrations with most of the points lying within FAC2 region. All independent variables have highly significant effects (*P* < 0.001) on NO_2__DG. Although GAM shows better performance than MLRM, MLRM are used more often by researchers due to the ease with which it can be applied and interpreted. MLRM provide a slope for each explanatory variable as it assumes a linear relationship, whereas in the case of GAM, the slope changes almost at every point (Fig. 11). In real-life situations especially in the case of air quality data, relationships are not always linear; therefore, GAM provide a better option for air quality modelling and display greater predictability as shown in this study. To explain this further, several plots are shown in Fig. 13 showing that the association between various air pollutants is not linear. To address the non-linear association, we need a non-linear model. GAM successfully addresses the non-linear association between various air pollutants and so performs better than a linear model. A demonstrative is shown in Fig. 13 (lower-right panel), where the value of *R*^2^ is 0.79 for GAM and 0.5 for LRM showing considerable difference in performance of the two models.

**Table 4:** Showing different statistical metrics for GAM

Response variable	Explanatory variable(s)	FAC2	RMSE	*R* ^2^	NMB	COE
NO_DG	NO_mean	0.98	2.80	0.17	0.014	0.101
NO_2__DG	NO_2__mean	0.80	10.06	0.16	0.012	0.048
NO_DG	NO_mean, NO_2__mean, NO_2__DG, WS, RH, Temp	0.53	9.89	0.70	0.008	0.50
NO_2__DG	NO_mean, NO_2__mean, NO_DG, WS, RH, Temp	0.95	3.91	0.83	0.005	0.614

### Further discussion of LCS

Castell et al. (2016) have evaluated the performance of the AQMesh sensors measuring gaseous air pollutants (e.g. NOx, CO and O_3_) and particulate matter (PM_10_ and PM_2.5_) in Oslo, Norway. They performed the evaluation both outdoors and under indoor laboratory conditions. They considered several types of emissions and environmental conditions such as roadside traffic and urban background over a 6-month period (April to September, 2015). Castell et al. (2016) concluded that good performance of the low-cost sensors in the laboratory does not imply similar performance when sited outdoors. Therefore, to reduce uncertainties, sensors must be calibrated in outdoor field locations. They also concluded that there is a lack of adequate outdoor testing of the sensors by the manufacturers before marketing such sensors, which can lead to poor performance and misleading data, which is of great concern, especially when members of the public use such instruments without scientific supervision to collect and interpret air quality data.

Borrego et al. (2016) compared the performance of several LCS with reference instruments from 13 to 27 October 2014 and reported that for measuring O_3_, AQMesh and NanoEnvi sensors had the lowest errors and higher coefficient of determination (*R*^2^ > 0.70), whereas ENEA Air-Sensors, ISAG and Cambridge SNAQ showed poor performance with *R*^2^ < 0.2. To measure the levels of NO_2_, Borrego et al. (2016) compared the performance of six platforms, where the highest correlation and lowest errors were shown by AQMesh, ECN Airbox and Cambridge University SNAQ with *R*^2^ > 0.80 and mean biased error (MBE) close to zero. In contrast, ENEA Air-Sensors and AUTh-ISAG AQ Microsensors demonstrated very poor correlation (*R*^2^ < 0.1). For measuring the levels of CO, AQMesh and Cambridge University SNAQ had the highest correlation (*R*^2^ > 0.80) with reference instruments, whereas the performance of the rest of the sensors was also satisfactory (*R*^2^ > 0.50) (Borrego et al. 2016). For monitoring NO, AQMesh and Cambridge University SNAQ were compared, where AQMesh showed better correlation (*R*^2^ = 0.80) than Cambridge University SNAQ (*R*^2^ = 0.30). For measuring PM_10_, all sensors showed poor correlation with reference instruments, with *R*^2^ = 0.36 being the highest which was observed with the ECN Airbox (Borrego et al. 2016). The ECN Airbox also showed the highest correlation (*R*^2^ = 0.27) with reference instruments for measuring PM_2.5_, the other sensors had lower *R*^2^-values.

Castell et al. (2017) compared the measurements from 24 AQMesh sensors against reference instruments and reported that the quality of the data obtained from the LCS were questionable. The performance of the sensors varied both spatially and temporally and was dependent on the atmospheric composition and meteorological conditions, such as temperature and relative humidity. Furthermore, Castell et al. (2017) reported that the performance varied from unit to unit; therefore, it is necessary to check the data quality of each pod separately before use. The sensors installed in the laboratory showed much stronger correlation (*R*^2^ > 0.95 for all pollutants) with reference instruments than those installed outdoors, where the average *R*^2^ values were 0.60, 0.86, 0.49, 0.54, 0.56 and 0.51 for CO, NO, NO_2_, O_3_, PM_10_ and PM_2.5_, respectively. Air quality data collected by means of LCS are suitable for promoting air quality awareness, general information and for highlighting air pollution hotpots; however, the data are not suitable for air quality compliance and research, especially for assessing health and environmental impacts of air pollution (Castell et al. 2017). Dongol (2015) has also concluded that air quality data collected by LCS cannot be used for air quality regulatory purposes and for other purposes where highly accurate data are required. Therefore, Lewis and Edwards (2016) state there is a need for further legislation to regulate the usability of data obtained from low-cost sensors.

Referring to the uncertainties in air quality data collected by LCS, Lewis and Edwards (2016) have commented that the recent introduction of these sensors for monitoring public exposure to air pollution are generating a large volume of data, which remain mostly untested, and therefore their quality is questionable and will create difficulty for air quality managers and planners in the future. Furthermore, Lewis and Edwards (2016) mentioned that these sensors show stability and sensitivity issues and that the sensors’ readings are subject to interference from other long-lived air pollutants, e.g. CO_2_ and H_2_ and prevailing meteorological conditions like relative humidity, temperature and wind speed. The lower-cost sensors perform better when air pollutant levels are high (Lewis and Edwards 2016). The lower-cost sensors have potential to measure air pollutant levels in places where traditional monitoring was not previously possible. They are portable, cheaper, and can provide much better spatial and temporal coverage in real-time, providing more localised and timely warnings to the public.

Lewis et al. (2016) have shown that one potential solution to reduce the uncertainties of air quality data obtained by using this class of sensors is by applying supervised machine learning techniques, such as the boosted regression tree (BRT) model. Spinelle et al. (2017) applied three approaches for calibrating the concentration of NO_2_, CO and CO_2_. The methods were linear regression, multiple linear regression and a supervised machine learning technique (artificial neural network). Using simple linear regression, only the reference concentration was used as an explanatory variable, whereas in the other models, relative humidity and temperature were also used. Supervised learning technique showed better performance than the other two models. The finding of this current study agrees with the above previous studies and show that the quality of NO_2_ concentrations measured by LCS can be much improved by applying supervised machine learning techniques based on GAM.

## Conclusions

LCS have the potential to contribute to real-time air quality monitoring networks installed to date as this type of sensors are cheap, compact, user-friendly and provide high-resolution spatiotemporal measurements of air pollutant concentrations. However, these sensors have limitations; therefore, the sensors require outdoor calibration and the data obtained from these sensors require further processing employing advanced statistical modelling approaches, such as GAM. In this paper, air pollutant data from ten Envirowatch E-MOTEs were compared with each other and with reference instruments. The sensors were able to capture the diurnal, weekly and annual cycles of air pollutant concentrations with some discrepancies. NO_2_ and CO showed stronger correlation between various sensors, where most of the correlation coefficients were greater than 0.9; however, NO showed relatively weaker correlation between the various sensor locations. NO_2_ concentrations showed very strong positive correlation between various sensors. Mostly, correlation coefficients (*r* values) were greater than 0.92. CO from different sensors also had *r* values mostly greater than 0.92; however, NO showed *r* value less than 0.5. Several linear and non-linear models were developed for sensor calibration and for predicting NO_2__DG and NO_DG concentrations using NO_mean and NO_2__mean and meteorological parameters as explanatory variables. GAM demonstrated better performance by exhibiting stronger similarity (e.g. greater correlation coefficient and FAC2 values) and lower error (e.g. weaker RMSE and NMB) between observed and modelled concentrations of NO and NO_2_. GAM were able to capture the non-linear association between various air pollutants and performed better than linear models. The best GAM developed for reproducing NO_2_ concentrations returned values of 0.95, 3.91, 0.81, 0.005, and 0.61 for factor of two (FAC2), root mean square error (RMSE), coefficient of determination (*R*^2^), normalised mean biased (NMB) and coefficient of efficiency (COE), respectively. Therefore, GAM are recommended for LCS calibration and for reproducing measured NO_2_. In the coming projects, we intend to deploy a more dense network of LCS in the whole city of Sheffield to collect high-resolution spatial and temporal air quality data. We also aim to improve experimental designs of the sensor network, test other sensor technologies and identify new calibration approaches for better performance in the future.
